# Kernel Temporal Differences for Neural Decoding

**DOI:** 10.1155/2015/481375

**Published:** 2015-03-17

**Authors:** Jihye Bae, Luis G. Sanchez Giraldo, Eric A. Pohlmeyer, Joseph T. Francis, Justin C. Sanchez, José C. Príncipe

**Affiliations:** ^1^Department of Electrical and Computer Engineering, University of Florida, Gainesville, FL 32611, USA; ^2^Department of Biomedical Engineering, University of Miami, Coral Gables, FL 33146, USA; ^3^Department of Physiology and Pharmacology, Robert F. Furchgott Center for Neural & Behavioral Science, SUNY Downstate Medical Center, Brooklyn, NY 11203, USA

## Abstract

We study the feasibility and capability of the kernel temporal difference (KTD)(*λ*) algorithm for neural decoding. KTD(*λ*) is an online, kernel-based learning algorithm, which has been introduced to estimate value functions in reinforcement learning. This algorithm combines kernel-based representations with the temporal difference approach to learning. One of our key observations is that by using strictly positive definite kernels, algorithm's convergence can be guaranteed for policy evaluation. The algorithm's nonlinear functional approximation capabilities are shown in both simulations of policy evaluation and neural decoding problems (policy improvement). KTD can handle high-dimensional neural states containing spatial-temporal information at a reasonable computational complexity allowing real-time applications. When the algorithm seeks a proper mapping between a monkey's neural states and desired positions of a computer cursor or a robot arm, in both open-loop and closed-loop experiments, it can effectively learn the neural state to action mapping. Finally, a visualization of the coadaptation process between the decoder and the subject shows the algorithm's capabilities in reinforcement learning brain machine interfaces.

## 1. Introduction

Research in brain machine interfaces (BMIs) is a multidisciplinary effort involving fields such as neurophysiology and engineering. Developments in this area have a wide range of applications, especially for subjects with neuromuscular disabilities, for whom BMIs may become a significant aid. Neural decoding of motor signals is one of the main tasks that needs to be executed by the BMI.

Ideas from system theory can be used to frame the decoding problem. Bypassing the body can be achieved by modelling the transfer function from brain activity to limb movement and utilizing the output of the properly trained model to control a robotic device to implement the intention of movement. The design of neural decoding systems has been approached using machine learning methods. In order to choose the appropriate learning method, factors such as learning speed and stability help in determining the usefulness of a particular method.

Reinforcement learning brain machine interfaces (RLBMI) [1] have been shown to be a promising avenue for practical implementations. Fast adaptation under changing environments and neural decoding capability of an agent have been shown in [2, 3] using the actor-critic paradigm. Adaptive classification of event-related potential (ERP) in electroencephalography (EEG) using RL in BMI was proposed in [4]. Moreover, partially observable Markov decision processes (POMDPs) have been applied in the agent to account for the uncertainty when decoding noisy brain signals [5]. In a RLBMI, a computer agent and a user in the environment cooperate and learn coadaptively. The decoder learns how to correctly translate neural states into action direction pairs that indicate the subject's intent. In the agent, the proper neural decoding of the motor signals is essential to control an external device that interacts with the physical environment.

However, to realize the advantages of RLBMIs in practice, there are several challenges that need to be addressed. The neural decoder must be able to handle high-dimensional neural states containing spatial-temporal information. The mapping from neural states to actions must be flexible enough to avoid making strong assumptions. Moreover, the computational complexity of the decoder should be reasonable such that real time implementations are feasible.

Temporal difference learning provides an efficient learning procedure that can be applied to reinforcement learning problems. In particular, TD(*λ*) [6] can be applied to approximate a value function that is utilized to compute an approximate solution to Bellman's equation. The algorithm allows incremental computation directly from new experience without having an associated model of the environment. This provides a means to efficiently handle high-dimensional states and actions by using an adaptive technique for function approximation that can be trained directly from the data. Also, because TD learning allows system updates directly from the sequence of states, online learning is possible without having a desired signal at all times.

Note that TD(*λ*) and its variants (least squares TD(*λ*) [7], recursive least squares TD [8], incremental least squares TD(*λ*) [9], Gradient TD [10], and linear TD with gradient correction [11]) have been mostly treated in the context of parametric linear function approximation. This can become a limiting factor in practical applications where little prior knowledge can be incorporated. Therefore, here, our interest focuses on a more general class of models with nonlinear capabilities. In particular, we adopt a kernel-based function approximation methodology.

Kernel methods are an appealing choice due to their elegant way of dealing with nonlinear function approximation problems. Unlike most of the nonlinear variants of TD algorithms which are prone to fall into local minima [12, 13], the kernel based algorithms have nonlinear approximation capabilities yet the cost function can be convex [14]. One of the major appeals of kernel methods is the ability to handle nonlinear operations on the data by an implicit mapping to the so called* feature space* (reproducing kernel Hilbert space (RKHS)) which is endowed with an inner product. A linear operation in the RKHS corresponds to a nonlinear operation in the input space. In addition, algorithms based on kernel methods are still reasonably easy to compute based on the kernel trick [14].

Temporal difference algorithms based on kernel expansions have shown superior performance in nonlinear approximation problems. The close relation between Gaussian processes and kernel recursive least squares was exploited in [15] to provide a Bayesian framework for temporal difference learning. Similar work using kernel-based least squares temporal difference learning with eligibilities (KLSTD(*λ*)) was introduced in [16]. Unlike the Gaussian process temporal difference algorithm (GPTD), KLSTD(*λ*) is not a probabilistic approach. The idea in KLSTD is to extend LSTD(*λ*) [7] using the concept of duality. However, the computational complexity of KLSTD(*λ*) per time update is *O*(*n* ^3^) which precludes its use for online learning.

An online kernel temporal difference learning algorithm called kernel temporal differences (KTD)(*λ*) was proposed in [17]. By using stochastic gradient updates, KTD(*λ*) reduces the computational complexity from *O*(*n* ^3^) to *O*(*n* ^2^). This reduction along with other capacity control mechanisms such as sparsification make real time implementations of KTD(*λ*) feasible.

Even though nonparametric techniques are inherently of growing structure, these techniques produce better solutions than any other simple linear function approximation methods. This has motivated work on methods that help overcome scalability issues such as the growing filter size [18]. In the context of kernel based TD algorithms, sparsification methods such as approximate linear dependence (ALD) [19] have been applied to GPTD [15] and KLSTD [20]. A Quantization approach proposed in [21] has been used in KTD(*λ*) [22]. In a similar flavor, the kernel distance based online sparsification method was proposed for a KTD algorithm in [23]. Note that ALD is *O*(*n* ^2^) complexity, whereas quantization and kernel distances are *O*(*n*). The main difference between the quantization approach and the kernel distance is the space where the distances are computed. Quantization approach uses criterion of input space distances whereas kernel distance computes them in the RKHS associated with the kernel [23].

In this paper, we investigate kernel temporal differences (KTD)(*λ*) [17] for neural decoding. We first show the advantages of using kernel methods. Namely, we show that convergence results of TD(*λ*) in policy evaluation carry over KTD(*λ*) when the kernel is strictly positive definite. Examples of the algorithm's capability for nonlinear function approximation are also presented. We apply the KTD algorithm to neural decoding in open-loop and closed-loop RLBMI experiments where the algorithm's ability to find proper neural state to action map is verified. In addition, the trade off between the value function estimation accuracy and computation complexity under growing filter size is studied. Finally, we provide visualizations of the coadaptation between the decoder and the subject highlighting the usefulness of KTD(*λ*) for reinforcement learning brain machine interfaces.

This paper starts with a general background on reinforcement learning which is given in Section 2.  Section 3 introduces the KTD algorithm and provides its convergence properties for policy evaluation. This algorithm is extended in Section 4 to policy improvement using *Q*-learning.  Section 5 introduces some of the kernel sparsification methods for the KTD algorithm that address the naturally growing structure of kernel adaptive algorithms.  Section 6 shows empirical results on simulations for policy evaluation, and Section 7 presents experimental results and comparisons to other methods in neural decoding using real data sets for both, open-loop and closed-loop RLBMI frameworks. Conclusions are provided in Section 8.

## 2. Reinforcement Learning Brain Machine Interfaces and Value Functions

**Figure 1:**
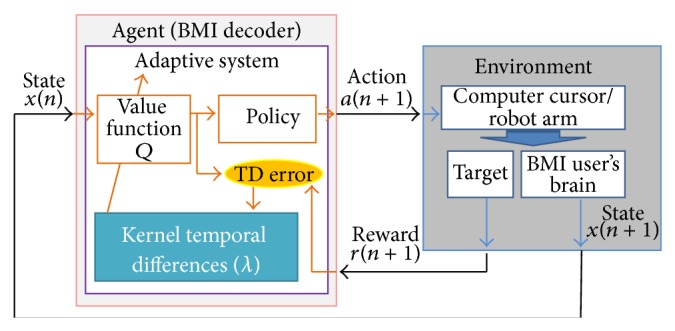
The decoding structure of reinforcment learning model in a brain machine interface using a *Q*-learning based function approximation algorithm.

In reinforcement learning brain machine interfaces (RLBMI), a neural decoder interacts with environment over time and adjusts its behavior to improve performance [1]. The controller in the BMI can be considered as a neural decoder, and the environment includes the BMI user (Figure 1).

Assuming the environment is a stochastic and stationary process that satisfies the Markov condition, it is possible to model the interaction between the learning agent and the environment as a Markov decision process (MDP). For the sake of simplicity, we assume the states and actions are discrete, but they can also be continuous.

At time step *n*, the decoder receives the representation of the user's neural state *x*(*n*) ∈ *𝒳* as input. According to this input, the decoder selects an action *a*(*n*) ∈ *𝒜* which causes the state of the external device to change, namely, the position of a cursor on a screen or a robot's arm position. Based on the updated position, the agent receives a reward *r*(*n* + 1) ∈ *ℛ*. At the same time, the updated position of the actuator will influence the user's subsequent neural states; that is, going from *x*(*n*) to *x*(*n* + 1) because of the visual feedback involved in the process. The new state *x*(*n* + 1) follows the state transition probability *𝒫*_*xx*′_^*a*^ given the action *a*(*n*) and the current state *x*(*n*). At the new state *x*(*n* + 1), the process repeats; the decoder takes an action *a*(*n* + 1), and this will result in a reward *r*(*n* + 2) and a state transition from *x*(*n* + 1) to *x*(*n* + 2). This process continues either indefinitely or until a terminal state is reached depending on the process.

Note that the user has no direct access to actions, and the decoder must interpret the user's brain activity correctly to facilitate the rewards. Also, both systems act symbiotically by sharing the external device to complete their tasks. Through iterations, both systems learn how to earn rewards based on their joint behavior. This is how the two intelligent systems (the decoder and the user) learn coadaptively, and the closed loop feedback is created. This coadaptation allows for continuous synergistic adaptation between the BMI decoder and the user even in changing environments [1].

The value function is a measure of long-term performance of an agent following a policy *π* starting from a state *x*(*n*). The state value function is defined as

(1) $$\begin{array}{c} V^{\pi }\left(x\left(n\right)\right)=E_{\pi }\left[R\left(n\right)\;|\;x\left(n\right)\right], \end{array}$$

and action value function is given by

(2) $$\begin{array}{c} Q^{\pi }\left(x\left(n\right),a\left(n\right)\right)=E_{\pi }\left[R\left(n\right)\;|\;x\left(n\right),a\left(n\right)\right], \end{array}$$

where **R**(*n*) is known as the return. Here, we apply a common choice for the return, the* infinite-horizon* discounted model

(3) $$\begin{array}{c} R\left(n\right)=\overset{\infty }{\underset{k=0}{\sum }}\gamma^{k}r\left(n+k+1\right),\;0<\gamma <1 \end{array}$$

that takes into account the rewards in the long run but weighs them with a discount factor to prevent the function from growing unbounded as *k* → *∞* and provides mathematical tractability [24]. Note that our goal is to find a policy *π* : *𝒳* → *𝒜* which maps a state *x*(*n*) to an action *a*(*n*). Estimating the value function is an essential step towards finding a proper policy.

## 3. Kernel Temporal Difference(*λ*)

In this section, we provide a brief introduction to kernel methods followed by the derivation of the KTD algorithm [17, 22]. One of the contributions of the present work is the convergence analysis of KTD(*λ*) presented at the end of this section.

### 3.1. Kernel Methods

Kernel methods are a family of algorithms for which input data are nonlinearly map to a high-dimensional feature space of vectors where linear operations are carried out. Let *𝒳* be a nonempty set. For a positive definite function *κ* : *𝒳* × *𝒳* → *ℛ* [14, 25], there exists a Hilbert space *ℋ* and a mapping *ϕ* : *𝒳* → *ℋ* such that

(4) $$\begin{array}{c} \kappa \left(x,y\right)=\langle \phi \left(x\right),\phi \left(y\right)\rangle . \end{array}$$

The inner product in the high-dimensional feature space can be calculated by evaluating the kernel function in the input space. Here, *ℋ* is called a reproducing kernel Hilbert space (RKHS), for which the following property holds,

(5) $$\begin{array}{c} f\left(x\right)=\langle f,\phi \left(x\right)\rangle =\langle f,\kappa \left(x,\cdot \right)\rangle ,\;\forall f\in H. \end{array}$$

The mapping implied by the use of the kernel function *κ* can also be understood through Mercer's Theorem [26]. The implicit map *ϕ* allows one to transform conventional linear algorithms in the feature space to nonlinear systems in the input space, and the kernel function *κ* provides an implicit way to compute inner products in the RKHS without explicitly dealing with the high-dimensional space.

### 3.2. Kernel Temporal Difference(*λ*)

In the multistep prediction problem, we consider a sequence of input-output pairs (*x*(1), *d*(1)), (*x*(2), *d*(2)),…, (*x*(*m*), *d*(*m*)), for which the desired output *d* is only available at time *m* + 1. Consequently, the system should produce a sequence of predictions *y*(1), *y*(2),…, *y*(*m*) based solely on the observed input sequences before it gets access to the desired response. In general, the predicted output is a function of all previous inputs *y*(*n*) = *f*(*x*(1), *x*(2),…, *x*(*n*)). Here, we assume that *y*(*n*) = *f*(*x*(*n*)) for simplicity, and let the function *f* belong to a RKHS *ℋ*.

In supervised learning, by treating the observed input sequence and the desired prediction as a sequence of pairs (*x*(1), *d*), (*x*(2), *d*),…, (*x*(*m*), *d*) and making *d*≜*y*(*m* + 1), we can obtain the updates of function *f* after the whole sequence of *m* inputs has been observed as

(6) f⟵f+∑n=1mΔfn

(7)  =f+η∑n=1md−fxnϕxn.

Here, Δ*f*_*n*_ = *η*[*d* − 〈*f*, *ϕ*(*x*(*n*))〉]*ϕ*(*x*(*n*)) are the instantaneous updates of the function *f* from input data based on the kernel expansion (5).

The key observation to extend the supervised learning approach to the TD method is that the difference between desired and predicted output at time *n* can be written as

(8) $$\begin{array}{c} d-y\left(n\right)=\overset{m}{\underset{k=n}{\sum }}\left(y\left(k+1\right)-y\left(k\right)\right), \end{array}$$

where *y*(*m* + 1)≜*d*. Using this expansion in terms of the differences between sequential predictions, we can update the system at each time step. By replacing the error *d* − *f*(*x*(*n*)) in (7) using the relation with temporal differences (8) and rearranging the equation as in [6], we obtain the following update:

(9) $$\begin{array}{c} f⟵f+\eta \overset{m}{\underset{n=1}{\sum }}\left[f\left(x\left(n+1\right)\right)-f\left(x\left(n\right)\right)\right]\overset{n}{\underset{k=1}{\sum }}\phi \left(x\left(k\right)\right). \end{array}$$

In this case, all predictions are used equally. Using exponential weighting on recency yields the following update rule:

(10) $$\begin{array}{c} f⟵f+\eta \overset{m}{\underset{n=1}{\sum }}\left[f\left(x\left(n+1\right)\right)-f\left(x\left(n\right)\right)\right]\overset{n}{\underset{k=1}{\sum }}\lambda^{n-k}\phi \left(x\left(k\right)\right). \end{array}$$

Here, *λ* represent an eligibility trace rate that is added to the averaging process over temporal differences to emphasize on the most recently observed states and to efficiently deal with delayed rewards.

The above update rule (10) is called kernel temporal difference (KTD)(*λ*) [17]. The difference between predictions of sequential inputs is called temporal difference (TD) error,

(11) $$\begin{array}{c} e_{\text{TD}}\left(n\right)=f\left(x\left(n+1\right)\right)-f\left(x\left(n\right)\right). \end{array}$$

Note that the temporal differences *f*(*x*(*n* + 1)) − *f*(*x*(*n*)) can be rewritten using the kernel expansions as 〈*f*, *ϕ*(*x*(*n* + 1))〉−〈*f*, *ϕ*(*x*(*n*))〉. This yields the instantaneous update of the function *f* as $\Delta \bar{f_{n}}=\eta \langle f,\phi (x(n+1))-\phi (x(n))\rangle \sum_{k=1}^{n}\lambda^{n-k}\phi (x(k))$. Using the RKHS properties, the evaluation of the function *f* at a given *x* can be calculated from the kernel expansion.

In reinforcement learning, the prediction *y*(*n*) = *f*(*x*(*n*)) can be considered as the value function (1) or (2). This is how the KTD algorithm provides a nonlinear function approximation to Bellman's equation. When the prediction *y*(*n*) represents the state value function, the TD error (11) is extended to the combination of a reward and sequential value function predictions. For instance, in the case of policy evaluation, the TD error is defined as

(12) $$\begin{array}{c} e_{\text{TD}}\left(n\right)=r\left(n+1\right)+\gamma V\left(x\left(n+1\right)\right)-V\left(x\left(n\right)\right). \end{array}$$

### 3.3. Convergence of Kernel Temporal Difference(*λ*)

It has been shown in [6, 27] that for an absorbing Markov chain, TD(*λ*) converges with probability 1 under certain conditions. Recall that the conventional TD algorithm assumes the function class to be linearly parametrized satisfying *y* = *w* ^*⊤*^ *x*. KTD(*λ*) can be viewed as a linear function approximation in the RKHS. Using this relation, convergence of KTD(*λ*) can be obtained as an extension of the convergence guarantees already established for TD(*λ*).

When *λ* = 1, by definition, the KTD(*λ* = 1) procedure is equivalent to the supervised learning method (7). KTD(1) yields the same per-sequence weight changes as the least square solution since (9) is derived directly from supervised learning by replacing the error term in (8). Thus, the convergence of KTD(1) can be established based on the convergence of its equivalent supervised learning formulation, which was proven in [25].

**Proposition 1 .**

The KLMS algorithm converges asymptotically in the mean sense to the optimal solution under the “small-step-size” condition.

**Theorem 2 .**

When the stepsize *η*_*n*_ satisfies *η*_*n*_ ≥ 0, ∑_*n*=1_^*∞*^ *η*_*n*_ = *∞* and ∑_*n*=1_^*∞*^ *η*_*n*_^2^ < *∞*, KTD(1) converges asymptotically in the mean sense to the least square solution.

**Proof**

Since by (8) the sequence of TD errors can be replaced by a multistep prediction with error *e*(*n*) = *d* − *y*(*n*), the result of Proposition 1 also applies to this case.

In the case of *λ* < 1, as shown by [27], the convergence of linear TD(*λ*) can be proved based on the ordinary differential equation (ODE) method introduced in [28]. This result can be easily extended to KTD(*λ*) as follows. Let us consider the Markov estimation problem as in [6]. An absorbing Markov chain can be described by the terminal and nonterminal sets of states *𝒯* and *𝒩*, transition probabilities *p*_*ij*_ between nonterminal states, the transition probabilities *s*_*ij*_ from nonterminal states to terminal states, the vectors *x*_*i*_ representing the nonterminal states, the expected terminal returns ${\bar{d}}_{j}$ from the *j*th terminal state, and the probabilities *μ*_*i*_ of starting at state *i*. Given an initial state *i* ∈ *𝒩*, an absorbing Markov chain generates an observation sequence of *m* vectors *x*_*i*_1__, *x*_*i*_2__,…, *x*_*i*_*m*__, where the last element *x*_*i*_*m*__ of the sequence corresponds to a terminal state *i*_*m*_ ∈ *𝒯*. The expected outcome $\bar{d}$ given a sequence starting at *i* ∈ *𝒩* is given by

(13)  e¯i∗ ≡Ed ∣ i

(14)    e¯i∗=∑j∈Tsijd¯j+∑j∈Npij∑k∈Tpjkd¯k+⋯

(15)    e¯i∗=∑k=0∞Qkhi=I−Q−1hi,

where [*x*]_*i*_ denotes the *i*th element of the array *x*, *Q* is the transition matrix with entries [*Q*]_*ij*_ = *p*_*ij*_ for *i*, *j* ∈ *𝒩*, and ${\left[h\right]}_{i}=\sum_{j\in 𝒯}s_{ij}{\bar{d}}_{j}$ for *i* ∈ *𝒩*. In linear TD(*λ*), a sequence of vectors *w*_1_, *w*_2_,…, is generated. Each one of these vectors *w*_*n*_ is generated after having a complete observation sequence; that is, a sequence staring at state *i* ∈ *𝒩* and ending at state *j* ∈ *𝒯* with the respective return *d*_*j*_. Similar to linear TD(*λ*), in KTD(*λ*) we have a sequence of functions *f*_1_, *f*_2_,…, (vectors in a RKHS) for which we can also write a linear update of the mean estimates of terminal return after *n* sequences have been observed. If *f*_*n*_ is the actual function estimate after sequence *n*, and ${\bar{f}}_{n+1}$ is the expected function estimate after the next sequence, we have that

(16) $$\begin{array}{c} {\bar{f}}_{n+1}\left(X\right)=f_{n}\left(X\right)+\eta_{n+1}H\left(f_{n}\left(X\right)-{\bar{e}}^{*}\right), \end{array}$$

where **H** = −**K** *D*[*I* − (1 − *λ*)*Q*(*I* − *λQ*)^−1^], [**K**]_*ij*_ = *κ*(*x*_*i*_, *x*_*j*_) with *i*, *j* ∈ *𝒩*, *D* is a diagonal matrix and [*D*]_*ii*_ the expected number of times the state *i* is visited during a sequence, and *f*_*n*_(*X*) is a column vector of function evaluations of the state representations such that [*f*_*n*_(*X*)]_*i*_ = *f*_*n*_(*x*_*i*_) = 〈*f*_*n*_, *ϕ*(*x*_*i*_)〉. Analogously to [27], the mean estimates in (16) converge appropriately if **H** has a full set of eigenvalues with negative real parts, for which we need **K** to be full rank. For the above to be true, it is required the set of vectors {*ϕ*(*x*_*i*_)}_*i*∈*𝒩*_ to be linearly independent in the RKHS. This is exactly the case when the kernel *κ* is strictly positive definite as shown in the following proposition.

**Proposition 3 .**

If *κ* : *𝒳* × *𝒳* → *ℛ* is a strictly positive definite kernel, for any finite set {*x*_*i*_}_*i*=1_^*N*^⊆*𝒳* of distinct elements, the set {*ϕ*(*x*_*i*_)} is linearly independent.

**Proof**

If *κ* is strictly positive definite, then ∑*α*_*i*_ *α*_*j*_ *κ*(*x*_*i*_, *x*_*j*_) > 0 for any set *x*_*i*_ where *x*_*i*_ ≠ *x*_*j*_, for all *i* ≠ *j*, and any *α*_*i*_ ∈ *ℛ* such that not all *α*_*i*_ = 0. Suppose there exists a set {*x*_*i*_} for which {*ϕ*(*x*_*i*_)} are not linearly independent. Then, there must be a set of coefficients *α*_*i*_ ∈ *ℛ* not all equal to zero such that ∑*α*_*i*_ *ϕ*(*x*_*i*_) = 0, which implies that ‖∑*α*_*i*_ *ϕ*(*x*_*i*_)‖^2^ = 0

(17) $$\begin{array}{c} 0=\sum \alpha_{i}\alpha_{j}\langle \phi \left(x_{i}\right),\phi \left(x_{j}\right)\rangle =\sum \alpha_{i}\alpha_{j}\kappa \left(x_{i},x_{j}\right), \end{array}$$

which contradicts the assumption.

The following Theorem is the resulting extension of* Theorem T* in [27] to KTD(*λ*).

**Theorem 4 .**

For any absorbing Markov chain, for any distribution of starting probailities *μ*_*i*_ such that there are not inaccessible states, for any outcome distributions with finite expected values ${\bar{d}}_{j}$, for any strictly positive definite kernel *κ*, and any set of observation vectors {*x*_*i*_,  *i* ∈ *𝒩*} such that *x*_*i*_ = *x*_*j*_ if and only if *i* = *j*, there exists an *ϵ* > 0 such that, if *η*_*n*_ = *η* where 0 < *η* < *ϵ* and for any initial function estimate, the predictions of KTD(*λ*) converge in expected value to the ideal predictions of (15). If *f*_*n*_ denotes the function estimate after experiencing *n* sequences, then

(18) $$\begin{array}{c} \underset{n\to \infty }{\lim}E\left[f_{n}\left(x_{i}\right)\right]=E\left[d\;|\;i\right]={\left[{\left(I-Q\right)}^{-1}h\right]}_{i},\;\forall i\in N. \end{array}$$

## 4. *Q*-Learning via Kernel Temporal Differences(*λ*)

Since the value function represents the expected cumulative rewards given a policy, the policy *π* is better than the policy *π*′ when the policy *π* gives greater expected return than the policy *π*′. In other words, *π* ≥ *π*′ if and only if *Q* ^*π*^(*x*, *a*) ≥ *Q* ^*π*′^(*x*, *a*) for all *x* ∈ *𝒳* and *a* ∈ *𝒜*. Therefore, the optimal action value function *Q* can be written as *Q* ^*^(*x*(*n*), *a*(*n*)) = max⁡_*π*_ *Q* ^*π*^(*x*(*n*), *a*(*n*)). The estimation can be done online. To maximize the expected reward *E*[*r*(*n* + 1)∣*x*(*n*), *a*(*n*), *x*(*n* + 1)], one-step *Q*-learning update was introduced in [29],

(19) Qxn,an⟵Q(x(n),a(n))  +ηrn+1+γmax⁡a Qxn+1,a    max⁡a−Qxn,an.

At time *n*, an action *a*(*n*) can be selected using methods such as *ϵ*-greedy or the Boltzmann distribution, which are popular for exploration and exploitation trade-off [30].

When we consider the prediction *y* as action value function *Q* ^*π*^ with respect to a policy *π*, KTD(*λ*) can approximate the value function ${\tilde{Q}}^{\pi }$ using a family of functions of the form

(20) $$\begin{array}{c} \tilde{Q}\left(x\left(n\right),a=i\right)=f\left(x\;|\;a=i\right)=\langle f,\phi \left(x\left(n\right)\right)\rangle . \end{array}$$

Here, $\tilde{Q}(x(n),a=i)$ denotes a state-action value given a state *x*(*n*) at time *n* and a discrete action *i*. Therefore, the update rule for *Q*-learning via kernel temporal difference (*Q*-KTD)(*λ*) can be written as

(21) f⟵f+η∑n=1mr(n+1)+γmax⁡a Q(x(n+1),a)      max⁡a−Q(x(n),a(n))∑k=1nλn−kϕ(x(k)).

We can see that the temporal difference (TD) error at time *n* includes reward and action value function terms. For single-step prediction problems (*m* = 1), (10) yields single updates for *Q*-KTD(*λ*) of the form:

(22) $$\begin{array}{c} Q_{i}\left(x\left(n\right)\right)=\eta \overset{n-1}{\underset{j=1}{\sum }}e_{\text{TD}i}\left(j\right)I_{k}\left(j\right)\kappa \langle x\left(n\right),x\left(j\right)\rangle . \end{array}$$

Here, *Q*_*i*_(*x*(*n*)) = *Q*(*x*(*n*), *a* = *i*) and *e*_TD*i*_(*n*) denotes the TD error defined as *e*_TD*i*_(*n*) = *r*_*i*_ + *γQ*_*ii*_(*x*(*n* + 1)) − *Q*_*i*_(*x*(*n*)), and *I*_*k*_(*n*) is an indicator vector of size determined by the number of outputs (actions). Only the *k*th entry of the vector is set to 1 and the other entries are set to 0. The selection of the action unit *k* at time *n* can be based on a greedy method. Therefore, only the weight (parameter vector) corresponding to the winning action gets updated. Recall that the reward *r*_*i*_ corresponds to the action selected by the current policy with input *x*(*n*) because it is assumed that this action causes the next input state *x*(*n* + 1).

**Figure 2:**
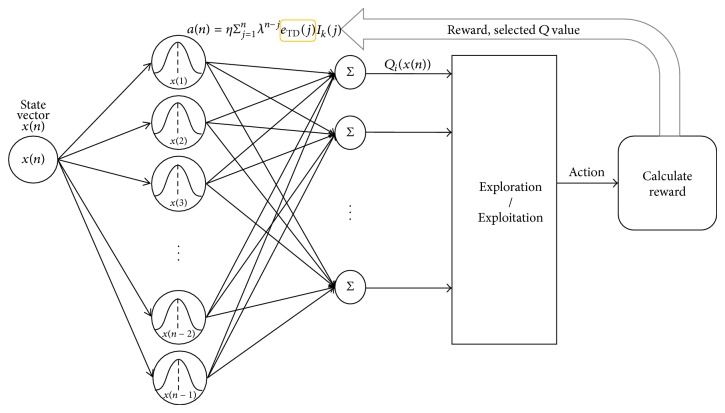
The structure of *Q*-learning via kernel temporal difference(*λ*).

The structure of *Q*-learning via KTD(0) is shown in Figure 2. The number of units (kernel evaluations) increases as more input data arrives. Each added unit is centered at the previous input locations *x*(1), *x*(2),…, *x*(*n* − 1).

In the reinforcement learning brain machine interface (RLBMI) paradigm, kernel temporal difference(*λ*) helps model the agent (see Figure 1). The action value function *Q* can be approximated using KTD(*λ*), for which the kernel based representations enhance the functional mapping capabilities of the system. Based on the estimated *Q* values, a policy decides a proper action. Note that the policy corresponds to the learning policy which changes over time in *Q*-learning.

## 5. Online Sparsification

One characteristic of nonparametric approaches is their inherently growing structure which is usually linear in the number of input data points. This rate of growth becomes prohibitive for practical applications that handle increasing amounts of incoming data over time. Various methods have been proposed to alleviate this problem (see [31] and references therein). These methods, known as kernel sparsification methods, can be applied to the KTD algorithm to control the growth of the terms in the function expansion, also known as filter size. Popular examples of kernel sparsification methods are the approximate linear dependence (ALD) [19], Surprise criterion [32], Quantization approach [21], and the kernel distance based method [23]. The main idea of sparsification is to only consider a reduced set of samples, called the dictionary, to represent the function of interest. The computational complexity of ALD is *O*(*d* ^2^), where *d* is the size of the dictionary. For the other methods mentioned above, the complexity is *O*(*d*).

Each of these methods has its own criterion to determine whether an incoming sample should be added to the current dictionary. The Surprise criterion [32] measures the subjective information of exemplar {*x*, *d*} with respect to a learning system Γ:

(23) $$\begin{array}{c} S_{\Gamma }\left(x,d\right)=-\ln p\left(x,d\;|\;\Gamma \right). \end{array}$$

Only samples with high values of Surprise are considered as candidates for the dictionary. In the case of the Quantization approach introduced in [21], the distance between a new input *x*(*n*) and the existing dictionary elements *C*(*n* − 1) is evaluated. The new input sample is added to the dictionary if the distance between the new input *x*(*n*) and the closest element in *C*(*n* − 1),

(24) $$\begin{array}{c} \underset{x_{i}\in C(n-1)}{\min}\left\|x\left(n\right)-x_{i}\right\|>\epsilon_{U}, \end{array}$$

is larger than the Quantization size *ϵ*_*U*_. Otherwise, the new input state *x*(*n*) is absorbed by the closest existing unit. Very similar to the quantization approach, the method presented in [23] applies a distance threshold criterion in the RKHS. The kernel distance based criterion given a state dictionary *D*(*n* − 1) adds a new unit when the new input state *x*(*n*) satisfies following condition;

(25) $$\begin{array}{c} \underset{x_{i}\in D(n-1)}{\min}{\left\|\phi (x(n))-\phi (x_{i})\right\|}^{2}>\mu_{1}. \end{array}$$

For some kernels such as Gaussian, the Quantization method and the kernel distance based criterion can be shown to be equivalent.

## 6. Simulations

Note that the KTD algorithm has been introduced for value function estimation. To evaluate the algorithm's nonlinear capability, we first examine the performance of the KTD(*λ*) in the problem of state value function estimation $\tilde{V}$ given a fixed policy *π*. We carry out experiments on a simple illustrative Markov chain initially described in [33]. This is a popular experiment involving an episodic task to test TD learning algorithms. The experiment is useful in illustrating linear as well as nonlinear functions of the state representations and shows how the state value function is estimated using the adaptive system.

### 6.1. Linear Case

**Figure 3:**
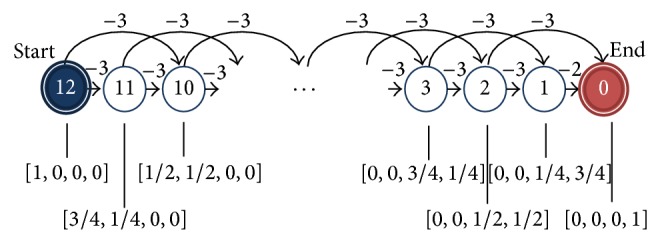
A 13-state Markov chain [33]. For states from 2 to 12, the state transition probabilities are 0.5 and the corresponding rewards are −3. State 1 has state transition probability of 1 to the terminal state 0 and a reward of −2. States 12, 8, 4, and 0 have the 4-dimensional state space representations [1,0, 0,0], [0,1, 0,0], [0,0, 1,0], and [0,0, 0,1], respectively. The representations of the other states are linear interpolations between the above vectors.

Even though we emphasize the capability of KTD(*λ*) as a nonlinear function approximator, under the appropriate kernel size, KTD(*λ*) should approximate linear functions on a region of interest as well. To test its efficacy, we observe the performance on a simple Markov chain (Figure 3). There are 13 states numbered from 12 to 0. Each trial starts at state 12 and terminates at state 0. Each state is represented by a 4-dimensional vector, and the rewards are assigned in such a way that the value function *V* is a linear function on the states; namely, *V* ^*^ takes the values [0, −2, −4,…, −22, −24] at states [0,1, 2,…, 11,12]. In the case of *V* = *w* ^*⊤*^ *x*, the optimal weights are *w* ^*^ = [−24, −16, −8,0].

To assess the performance, the updated estimate of the state value function $\tilde{V}(x)$ is compared to the optimal value function *V* ^*^ at the end of each trial. This is done by computing the RMS error of the value function over all states

(26) $$\begin{array}{c} \text{RMS}=\sqrt{\frac{1}{n}\underset{x\in X}{\sum }{\left(V^{*}(x)-\tilde{V}(x)\right)}^{2}}, \end{array}$$

where *n* is the number of states, *n* = 13.

Stepsize scheduling is applied as follows:

(27) $$\begin{array}{c} \eta \left(n\right)=\eta_{0}\frac{a_{0}+1}{a_{0}+n},\;\text{where}\;n=1,2,\ldots , \end{array}$$

where *η*_0_ is the initial stepsize, and *a*_0_ is the annealing factor which controls how fast the stepsize decreases. In this experiment, *a*_0_ = 100 is applied. Furthermore, we assume that the policy *π* is guaranteed to terminate, which means that the value function *V* ^*π*^ is well-behaved without using a discount factor *γ* in (3), that is, *γ* = 1.

In KTD(*λ*), we employ the Gaussian kernel:

(28) $$\begin{array}{c} \kappa \left(x\left(i\right),x\left(j\right)\right)=\exp\left(\frac{-{\left\|x(i)-x(j)\right\|}^{2}}{2h^{2}}\right), \end{array}$$

which is a universal kernel commonly encountered in practice. To find the optimal kernel size, we fix all the other free parameters around median values, *λ* = 0.4 and *η*_0_ = 0.5, and the average RMS error over 10 Monte Carlo runs is compared. For this specific experiment, smaller kernel sizes yield better performance since the state representations are finite. However, in general, applying too small kernel sizes leads to over-fitting and slow learning. In particular, choosing a very small kernel makes the algorithm behave very similar to the table look up method. Thus, we choose the kernel size *h* = 0.2 to be the largest kernel size for which we obtain similar mean RMS values as for smaller kernel sizes.

**Figure 4:**
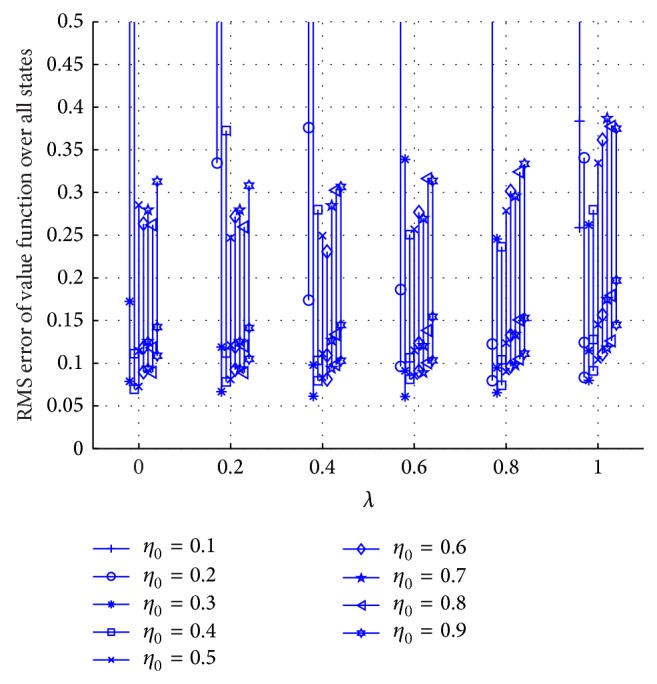
Performance comparison over different combinations of eligibility trace rates *λ* and initial step sizes *η*_0_ in KTD(*λ*) with *h* = 0.2. The vertical line segments contain the mean RMS value after 100 trials (top marker), 500 trials (middle marker), and 1000 trials (bottom marker).

After fixing the kernel size to *h* = 0.2, the experimental evaluation of different combinations of eligibility trace rates *λ* and initial step sizes *η*_0_ are observed.  Figure 4 shows the average performance over 10 Monte Carlo runs for 1000 trials.

All *λ* values with optimal stepsize show good approximation to *V* ^*^ after 1000 trials. Notice that KTD(*λ* = 0) shows slightly better performance than KTD(*λ* = 1). This may be attributed to the local nature of KTD when using the Gaussian kernel. In addition, varying the stepsize has a relatively small effect on KTD(*λ*). The Gaussian kernel as well as other shift-invariant kernels provide an implicit normalized update rule which is known to be less sensitive to stepsize. Based on Figure 4, the optimal eligibility trace rate and initial stepsize value *λ* = 0.6 and *η*_0_ = 0.3 are selected for KTD with kernel size *h* = 0.2.

**Figure 5:**
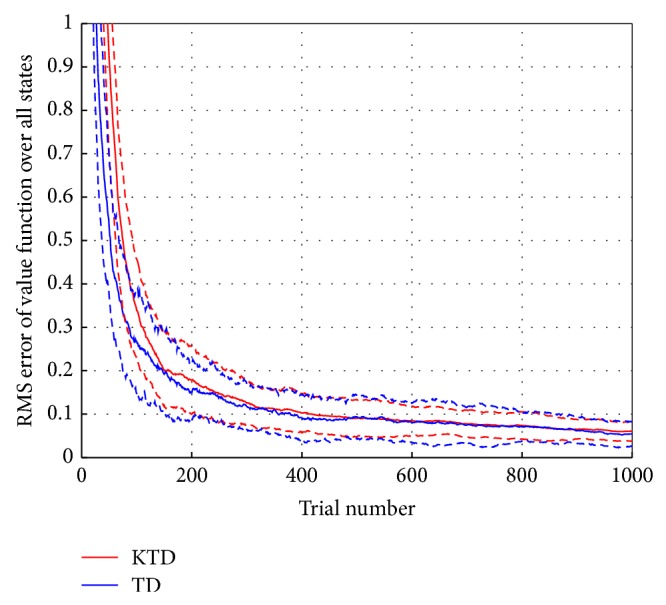
Learning curve of KTD(*λ*) and TD(*λ*). The solid line shows the mean RMS error, and the dashed line shows the +/− standard deviations over 50 Monte Carlo runs.

The learning curve of KTD(*λ*) is compared to the conventional TD algorithm, TD(*λ*). The optimal parameters employed in both algorithms are based on the experimental evaluation. In TD(*λ*), *λ* = 1 and *η*_0_ = 0.1 are applied. The RMS error is averaged over 50 Monte Carlo runs for 1000 trials. Comparative learning curves are given in Figure 5.

In this experiment, we confirm the ability of KTD(*λ*) to handle the function approximation problem when the fixed policy yields a state value function that is linear in the state representation. Both algorithms reach the mean RMS value of around 0.06. As we expected, TD(*λ*) converges faster to the optimal solution because of the linear nature of the problem. KTD(*λ*) converges slower than TD(*λ*), but it is also able to approximate the value function properly. In this sense, the KTD algorithm is open to wider class of problems than its linear counterpart.

### 6.2. Nonlinear Case

Previous section show the performances of KTD(*λ*) on the problem of estimating a state value function, which is a linear function of the given state representation. The same problem can be turned into a nonlinear one by modifying the reward values in the chain such that the resulting state value function *V* ^*^ is no longer a linear function of the states.

**Figure 6:**
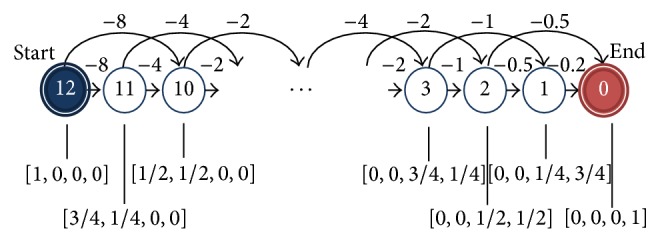
A 13-state Markov chain. In states from 2 to 12, each state transition has probability 0.5, and state 1 has transition probability 1 to the absorbing state 0. Note that optimal state value functions can be represented as a nonlinear function of the states, and corresponding reward values are assigned to each state.

The number of states and the state representations remain the same as in the previous section. However, the optimal value function *V* ^*^ becomes nonlinear with respect to the representation of the states; namely, *V* ^*^ = [0 −0.2−0.6−1.4−3 −6.2 −12.6 −13.4 −13.5−14.45 −15.975 −19.2125 −25.5938] for states 0 to 12. This implies that the reward values for each state are different from the ones given for the linear case (Figure 6).

**Figure 7:**
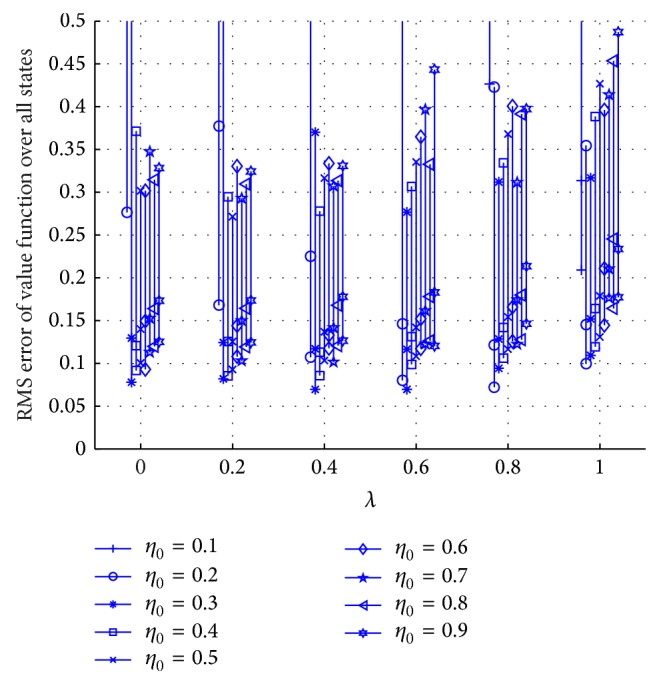
Performance comparison over different combinations of *λ* and the initial stepsize *η*_0_ in KTD(*λ*) with *h* = 0.2. The plotted segment is the mean RMS value after 100 trials (top segment), 500 trials (middle segment), and 1000 trials (bottom segment).

Again, to evaluate the performance, after each trial is completed, the estimated state value $\tilde{V}$ is compared to the optimal state value *V* ^*^ using RMS error (26). For KTD(*λ*), the Gaussian kernel (28) is applied and kernel size *h* = 0.2 is chosen.  Figure 7 shows the average RMS error over 10 Monte Carlo runs for 1000 trials.

The combination of *λ* = 0.4 and *η*_0_ = 0.3 shows the best performance, but the *λ* = 0 case also shows good performances. Unlike TD(*λ*) [6], there is no dominant value for *λ* in KTD(*λ*). Recall that it has been proved that convergence is guaranteed for linearly independent representations of the states, which is automatically fulfilled in KTD(*λ*) when the kernel is strictly positive definite. Therefore, the differences are rather due to the convergence speed controlled by the interaction between the step size and the elegibilty trace.

**Figure 8:**
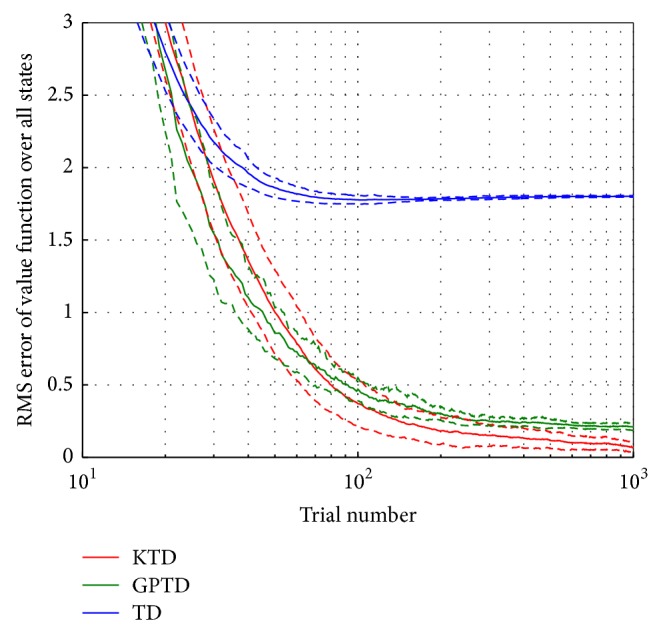
Learning curves of KTD(*λ*), GPTD, and TD(*λ*). The solid lines show the mean RMS error, and the dashed lines represent the (+/−) standard deviation over 50 Monte Carlo runs.

The average RMS error over 50 Monte Carlo runs is compared with Gaussian process temporal difference (GPTD) [15] and TD(*λ*) in Figure 8. The purpose of GPTD implementation is to have comparison among kernelized value function approximations. Here, the applied optimal parameters for KTD(*λ*) are *λ* = 0.4, *η*_0_ = 0.3, and *h* = 0.2, for GPTD, *λ* = 1, *σ* ^2^ = 0.5, and *h* = 0.2, and for TD(*λ*), *λ* = 0.8 and *η*_0_ = 0.1.

The linear function approximation, TD(*λ*) (blue line), cannot estimate the optimal state values. KTD(*λ*) outperforms the linear algorithm as expected since the Gaussian kernel is strictly positive definite. GPTD also learns the target state values, but the system fails to reach as low error values as KTD. GPTD is sensitive to the selection of the covariance value in the noise, *σ* ^2^; if the value is small, the system becomes unstable, and larger values cause the the learning to slow down. GPTD models the residuals, the difference between expected return and actual return, as a Gaussian process. This assumption does not hold true for the Markov chain in Figure 6. As we can observe in Figure 8, KTD(*λ*) reaches to the mean value around 0.07, and the mean value of GPTD and TD(*λ*) are around 0.2 and 1.8, respectively.

In the synthetic examples, we presented experimental results to approximate the state value function under a fixed policy. We observed that KTD(*λ*) performs well on both linear and nonlinear function approximation problems. In addition, in the previous section, we showed how the linear independence of the input state representations can affect the performance of algorithms. The use of strictly positive definite kernels in KTD(*λ*) implies the linear independence condition, and thus this algorithm converges for all *λ* ∈ [0,1]. In the following section, we will apply the extended KTD algorithm to estimate the action value function which can be employed in finding a proper control policy for RLBMI tasks.

## 7. Experimental Results on Neural Decoding

In our RLBMI experiments, we map the monkey's neural signal to action-directions (computer cursor/robot arm position). The agent starts at a naive state, but the subject has been trained to receive rewards from the environment. Once it reaches the assigned target, the system and the subject earn a reward, and the agent updates its neural state decoder. Through iteration, the agent learns how to correctly translate neural states into action-directions.

### 7.1. Open-Loop RLBMI

In open-loop RLBMI experiments, the output of the agent does not directly change the state of the environment because this is done with prerecorded data. The external device is updated based only on the actual monkey's physical response. In this sense, we only consider the monkey's neural state from successful trials to train the agent. The goal of these experiments is to evaluate the system's capability to predict the proper state to action mapping based on the monkey's neural states and to assess the viability of further closed-loop experiments.

#### 7.1.1. Environment

The data employed in these experiments is provided by SUNY Downstate Medical Center. A female bonnet macaque is trained for a center-out reaching task allowing 8 action-directions. After the subject attains about 80% success rate, microelectrode arrays are implanted in the motor cortex (M1). Animal surgery is performed under the Institutional Animal Care and Use Committee (IACUC) regulations and assisted by the Division of Laboratory Animal Resources (DLAT) at SUNY Downstate Medical Center.

From 96-channel recordings, a set of 185 units are obtained after sorting. The neural states are represented by the firing rates of each unit on 100 ms window. There is a set of 8 possible targets and action directions. Every trial starts at the center point, and the distance from the center to each target is 4 cm; anything within a radius of 1 cm from the target point is considered as a valid reach.

#### 7.1.2. Agent

In the agent, *Q*-learning via kernel temporal difference (*Q*-KTD)(*λ*) is applied to neural decoding. For *Q*-KTD(*λ*), we employ the Gaussian kernel (28). After the neural states are preprocessed by normalizing their dynamic range to lie between −1 and 1, they are input to the system. Based on the preprocessed neural states, the system predicts which direction the computer cursor will move. Each output unit represents one of the 8 possible directions, and among the 8 outputs one action is selected by the *ϵ*-greedy method [34]. The action corresponding to the unit with the highest *Q* value gets selected with probability 1 − *ϵ*. Otherwise, any other action is selected at random. The performance is evaluated by checking whether the updated position reaches the assigned target, and depending on the updated position, a reward value is assigned to the system.

#### 7.1.3. Results on Single Step Tasks

Here, the targets should be reached within a single step; rewards from the environment are received after a single step, and one action is performed by the agent per trial. The assignment of reward is based on the 1-0 distance to the target, that is, dist⁡(*x*, *d*) = 0 if *x* = *d*, and dist⁡(*x*, *d*) = 1, otherwise. Once the cursor reaches the assigned target, the agent gets a positive reward +0.6, else it receives negative reward −0.6 [35]. Exploration rate *ϵ* = 0.01 and discount factor *γ* = 0.9 are applied. Also, we consider *λ* = 0 since our experiment performs single step updates per trial. In this experiment, the firing rates of the 185 units on 100 ms windows are time-embedded using 6th order tap delay. This creates a representation space where each state is a vector with 1295 dimensions.

**Figure 9:**
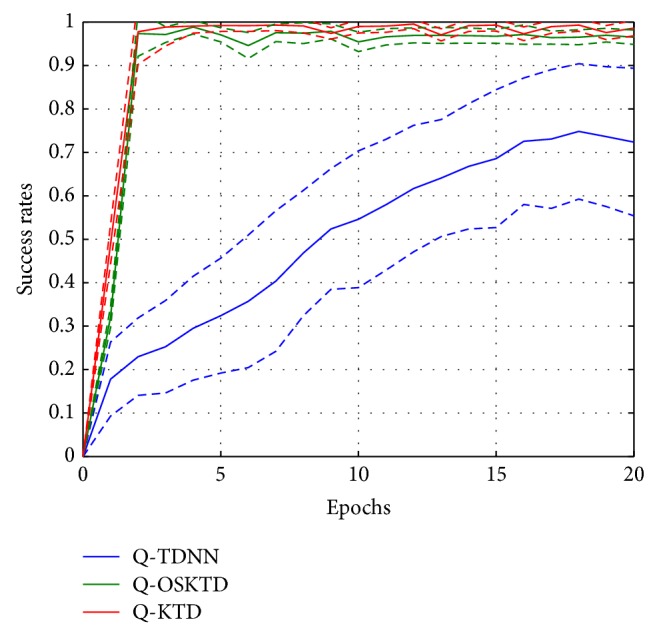
The comparison of average learning curves from 50 Monte Carlo runs among *Q*-TDNN, *Q*-OSKTD, and *Q*-KTD. Solid lines show the mean success rates and the dashed lines show the confidence interval based on one standard deviation.

We start with the simplest version of the problem by considering only 2-targets (right and left). The total number of trials is 43 for the 2-targets. For *Q*-KTD, the kernel size *h* is heuristically chosen based on the distribution of the mean squared distances between pairs of input states; let *s* = *E*[‖*x*_*i*_ − *x*_*j*_‖^2^], then $h=\sqrt{s/2}$. For this particular data set, the above heuristic gives a kernel size *h* = 7. The stepsize *η* = 0.3 is selected based on the stability bound that was derived for the kernel least mean square algorithm [25],

(29) $$\begin{array}{c} \eta <\frac{N}{\mathrm{tr}\left[G_{\phi }\right]}=\frac{N}{\sum_{j=1}^{N}\kappa \left(x\left(j\right),x\left(j\right)\right)}=1, \end{array}$$

where *G*_*ϕ*_ is the gram matrix. After 43 trials, we count the number of trials which received a positive reward, and the success rate is averaged over 50 Monte Carlo runs. The performance of the *Q*-KTD algorithm is compared with *Q*-learning via time delayed neural net (*Q*-TDNN) and the online selective kernel-based temporal difference learning algorithm (*Q*-OSKTD) [23] in Figure 9. Note that TDNN is a conventional approach to function approximation and has already been applied to RLBMI experiments for neural decoding [1, 2]. OSKTD is a kernel-based temporal difference algorithm emphasizing on the online sparsifications.

Both *Q*-KTD and *Q*-OSKTD reach around 100% success rate after 2 epochs. In contrast, the average success rate of *Q*-TDNN slowly increases yet never reaches the same performance as *Q*-KTD. In the case of *Q*-OSKTD, the value function updates require one more parameter *μ*_2_ to decide the subspace. To validate the algorithm's capability to estimate proper policy, we set the sparsified dictionary as the same size as the number of sample observations. In *Q*-OSKTD, we observed that the subspace selection parameter plays an important role in terms of the speed of learning. It turns out that for the above experiment, smaller subspaces allow faster learning. In the extreme case of *Q*-OSKTD, where only the current state is affected, the updates become equivalent to the update rule of *Q*-KTD.

Since all the experimental parameters are fixed over 50 Monte Carlo runs, the confidence interval for *Q*-KTD can be simply associated with the random effects introduced by the *ϵ*-greedy method employed for action selection with exploration, thus, the narrow interval. However, with *Q*-TDNN a larger variation of performance is observed, which shows how the initialization, due to local minima, influences the success of learning; it is observed that *Q*-TDNN is able to approximate the *Q*-KTD performance, but most of the times, the system falls on local minima. This highlights one of the advantages of KTD compared to TDNN, which is the insensitivity to initialization.

Table 1 shows average success rates over 50 Monte Carlo runs with respect to different number of targets. The first row corresponds to the mean success rates displayed on Figure 9 (red solid line). This is included in the Table 1 to ease comparison with 4 and 8-target experiments. The 4 target task involves reaching right, up, left and down positions from the center. Note that in all tasks, 8 directions are allowed at each step. The standard deviation of each epoch is around 0.02.

**Figure 10:**
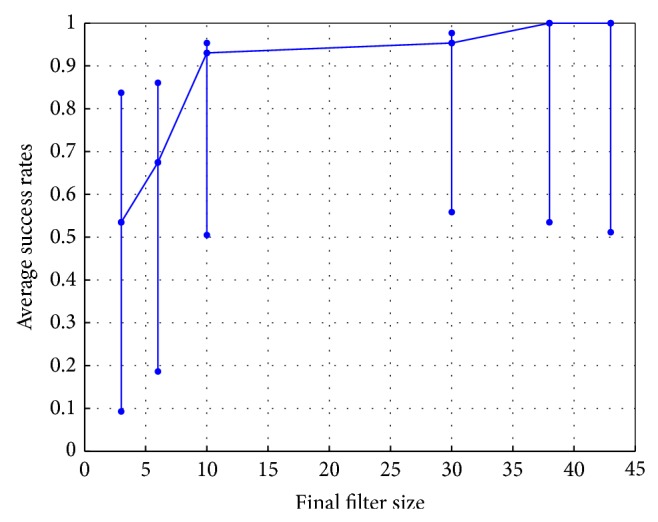
Average success rates over 50 Monte Carlo runs with respect to different filter sizes. The vertical line segments are the mean success rates after 1 epoch (bottom markers), 2 epochs (middle markers), and 20 epochs (top markers).

One characteristic of nonparametric approaches is the growing filter structure. Here, we observe how filter size influences the overall performance in *Q*-KTD by applying Surprise criterion [32] and Quantization [21] methods. In the case of the 2-target center-out reaching task, we should expect the filter size to become as large as 861 units after 20 epochs without any control of the filter size. Using the Surprise criterion, the filter size can be reduced to 87 centers with acceptable performance. However, Quantization allows the filter size to be reduced to 10 units while maintaining performance above 90% for success rates.  Figure 10 shows the effect of filter size in the 2-target experiment using the Quantization approach. For filter sizes as small as 10 units, the average success rates remain stable. With 10 units, the algorithm shows similar learning speed to the linearly growing filter size, with success rates above 90%. Note that quantization limits the capacity of the kernel filter since less units than samples are employed and thus it helps to avoid over-fitting.

In the 2-target center-out reaching task, quantized *Q*-KTD shows satisfactory results in terms of initialization and computational cost. Further analysis of *Q*-KTD is conducted on a larger number of targets. We increase the number of targets from 2 to 8. All experimental parameters are kept the same as for the 2-target experiment. The only change is step-size *η* = 0.5. The 178 trials are applied for the 8-target reaching task.

**Figure 11:**
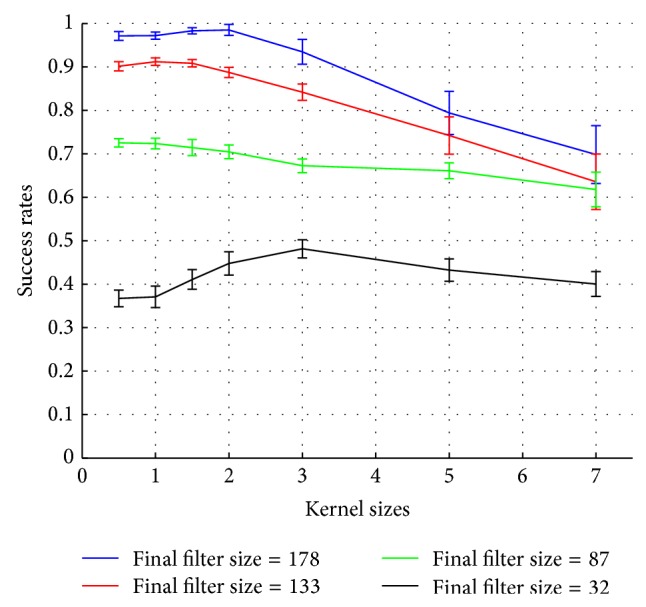
The effect of filter size control on 8-target single-step center-out reaching task. The average success rates are computed over 50 Monte Carlo runs after the 10th epoch.

To gain more insight about the algorithm, we observe the interplay between Quantization size *ϵ*_*U*_ and kernel size *h*. Based on the distribution of squared distances between pairs of input states, various kernel sizes (*h* = 0.5,1, 1.5,2, 3,5, 7) and Quantization sizes (*ϵ*_*U*_ = 1,110,120,130) are considered. The corresponding success rates for final filter sizes of 178, 133, 87, and 32 are displayed in Figure 11.

With a final filter size of 178 (blue line), the success rates are superior to any other filter sizes for every kernel sizes tested, since it contains all input information. Especially for small kernel sizes (*h* ≤ 2), success rates above 96% are observed. Moreover, note that even after reduction of the state information (red line), the system still produces acceptable success rates for kernel sizes ranging from 0.5 to 2 (around 90% success rates).

Among the best performing kernel sizes, we favor the largest one since it provides better generalization guarantees. In this sense, a kernel size *h* = 2 can be selected since this is the largest kernel size that considerably reduces the filter size and yields a neural state to action mapping that performs well (around 90% of success rates). In the case of kernel size *h* = 2 with final filter size of 178, the system reaches 100% success rates after 6 epochs with a maximum variance of 4%. As we can see from the number of units, higher representation capacity is required to obtain the desired performance as the task becomes more complex. Nevertheless, results on the 8-target center-out reaching task show that the method can effectively learn the brain state-action mapping for this task with a reasonable complexity.

#### 7.1.4. Results on Multistep Tasks

**Figure 12:**
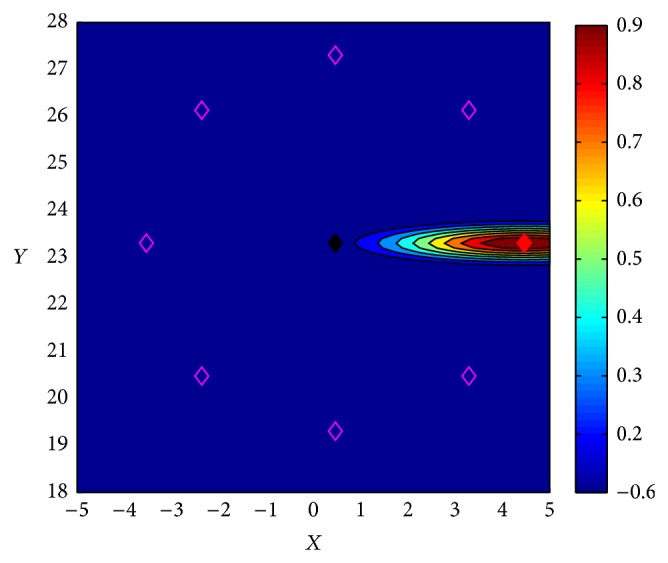
Reward distribution for right target. The black diamond is the initial position, and the purple diamond shows the possible directions including the assigned target direction (red diamond).

Here, we develop a more realistic scenario; we extend the task to multistep and multitarget experiments. This case allows us to explore the role of the eligibility traces in *Q*-KTD(*λ*). The price paid for this extension is that now, the eligibility trace rate *λ* selection needs to be carried out according to the best observed performance. Testing based on the same experimental set up employed for the single step task, that is, a discrete reward value is assigned at the target, causes extremely slow learning since not enough guidance is given. The system requires long periods of exploration until it actually reaches the target. Therefore, we employ a continuous reward distribution around the selected target defined by the following expression:

(30) $$\begin{array}{c} r\left(s\right)=\left\{\begin{array}{cc} p_{\text{reward}}G\left(s\right) & \text{if}\;G\left(s\right)>0.1,\\ n_{\text{reward}} & \text{if}\;G\left(s\right)\leq 0.1, \end{array}\right. \end{array}$$

where *G*(*s*) = exp⁡[(*s* − ***μ***)^*⊤*^ **C**_*θ*_^−1^(*s* − ***μ***)], *s* ∈ *ℛ* ^2^ is the position of the cursor, *p*_reward_ = 1, and *n*_reward_ = −0.6. The mean vector ***μ*** corresponds to the selected target location and the covariance matrix,

(31) $$\begin{array}{c} C_{\theta }=R_{\theta }\left(\begin{array}{cc} 7.5 & 0\\ 0 & 0.1 \end{array}\right)R_{\theta }^{⊤},\;\;R_{\theta }=\left(\begin{array}{cc} \cos\theta & \sin\theta \\ -\sin\theta & \cos\theta \end{array}\right), \end{array}$$

which depends on the angle *θ* of the selected target as follows: for target index one and five, the angle is 0, two and six are for −*π*/4, three and seven are for *π*/2, and four and eight are for *π*/4. (Here, the target indexes follow the location depicted on Figure  6 in [22].) Figure 12 shows the reward distribution for target index one. The same form of distribution is applied to the other directions centred at the assigned target point.

**Figure 13:**
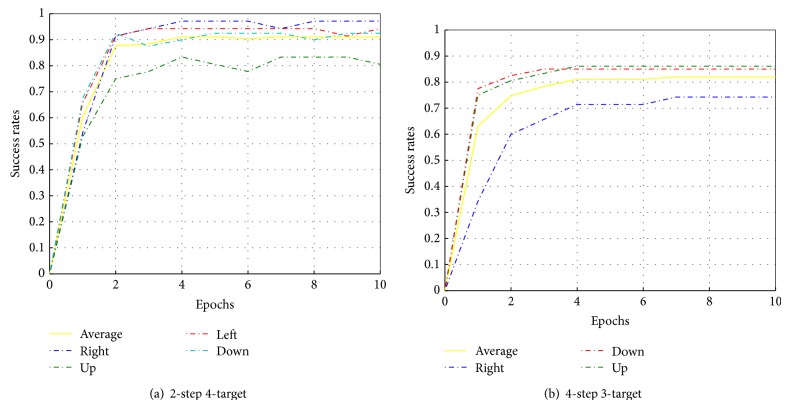
The learning curves for multistep multitarget tasks.

Once the system reaches the assigned target, the system earns a maximum reward of +1 and receives partial rewards according to (30) during the approaching stage. When the system earns the maximum reward, the trial is classified as a successful trial. The maximum number of steps per trial is limited such that the cursor must approach the target in a straight line trajectory. Here, we also control the complexity of the task by allowing different number of targets and steps. Namely, 2-step 4-target (right, up, left, and down) and 4-step 3-target (right, up, and down) experiments are performed. Increasing the number of steps per trial amounts to making smaller jumps according to each action. After each epoch, the number of successful trials is counted for each target direction.  Figure 13 shows the learning curves for each target and the average success rates.

Larger number of steps results in lower success rates. However, the two cases (two and four steps) obtain an average success rate above 60% for 1 epoch. The performances show all directions can achieve success rates above 70% after convergence, which encourage the application of the algorithm to online scenarios.

### 7.2. Closed-Loop RLBMI Experiments

In closed loop RLBMI experiments, the behavioral task is a reaching task using a robotic arm. The decoder controls the robot arm's action direction by predicting the monkey's intent based on its neuronal activity. If the robot arm reaches the assigned target, a reward is given to both the monkey (food reward) and the decoder (positive value). Notice that the two intelligent systems learn coadaptively to accomplish the goal. These experiments are conducted in cooperation with the Neuroprosthetics Research Group at the University of Miami. The performance is evaluated in terms of task completion accuracy and speed. Furthermore, we provide a methodology to tease apart the influence of each one of the systems of the RLBMI in the overall performance.

#### 7.2.1. Environment

During pretraining, a marmoset monkey was trained to perform a target reaching task, namely, moving a robot arm to two spatial locations denoted as A trial and B trial. The monkey was taught to associate changes in motor activity during A trials and produce static motor responses during B trials. Once a target is assigned, a beep signals the start of the trial. To control the robot during the user training phase, the monkey is required to steadily place its hand on a touch pad for 700~1200 ms. This action produces a* go* beep that is followed by the activation of one of the two target LEDs (A trial: red light for left direction or B trial: green light for right direction). The robot arm goes to a* home position*, namely, the center position between the two targets. Its gripper shows an object (food reward such as waxworm or marshmallow for A trial and undesirable object (wooden bead) for B trial). To move the robot to the A location, the monkey needed to reach out and touch a sensor within 2000 ms, and to make the robot reach to the B target, the monkey needed to keep its arm motionless on the touch pad for 2500 ms. When the monkey successfully moved the robot to the correct target, the target LEDs would blink and the monkey would receive a food reward (for both the A and B targets).

After the monkey is trained to perform the assigned task properly, a microelectrode array (16-channel tungsten microelectrode arrays, Tucker Davis Technologies, FL) is surgically implanted under isoflurane anesthesia and sterile conditions. Neural states from the motor cortex (M1) are recorded. These neural states become the inputs to the neural decoder. All surgical and animal care procedures were consistent with the National Research Council Guide for the Care and Use of Laboratory Animals and were approved by the University of Miami Institutional Animal Care and Use Committee.

In the closed-loop experiments, after the initial holding time that produces the* go* beep, the robotic arm's position is updated based solely on the monkey's neural states. Differently from the user pretraining sessions, the monkey is not required to perform any movement. During the real-time experiment, 14 neurons are obtained from 10 electrodes. The neural states are represented by the firing rates on a 2 sec window following the* go* signal.

#### 7.2.2. Agent

For the BMI decoder, we use *Q*-learning via kernel Temporal Differences (*Q*-KTD)(*λ*). One big difference between open-loop and closed-loop applications is the amount of accessible data; in the closed-loop experiments, we can only get information about the neural states that have been observed up to the present. However, in the previous offline experiments, normalization and kernel selection were conducted offline based on the entire data set. It is not possible to apply the same method to the online setting since we only have information about the input states up to the present time. Normalization is a scaling procedure that interplays with the choice of the kernel size. Proper selection of the kernel size brings proper scaling to the data. Thus, in contrast to the previous open-loop experiments, normalization of the input neural states is not applied, and the kernel size is automatically selected given the inputs.

The Gaussian kernel (28) is employed, and the kernel size *h* is automatically selected based on the history of inputs. Note that in the closed-loop experiments, the dynamic range of states varies from experiment to experiment. Consequently, the kernel size needs to be re-adjusted each time a new experiment takes place, and it cannot be determined beforehand. At each time, the distances between the current state and the previously observed states are computed to obtain the output values, $\tilde{Q}$ in this case. Therefore, we use the distance values to select the kernel size as follows:

(32) $$\begin{array}{c} h_{\text{temp}}\left(n\right)=\sqrt{\frac{1}{2\left(n-1\right)}\overset{n-1}{\underset{i=1}{\sum }}{\left\|x\left(i\right)-x\left(n\right)\right\|}^{2}},\\ h\left(n\right)=\frac{1}{n}\left[\overset{n-1}{\underset{i=1}{\sum }}h\left(i\right)+h_{\text{temp}}\left(n\right)\right]. \end{array}$$

Using the squared distance between pairs of previously seen input states, we can obtain an estimate of the mean distance. This value is also averaged along with past kernel sizes to obtain the current kernel size.

Moreover, we consider *γ* = 1 and *λ* = 0 since our experiments perform single step trials. Stepsize *η* = 0.5 is applied. The output represents the 2 possible directions (left and right), and the robot arm moves based on the estimated output from the decoder.

#### 7.2.3. Results

The overall performance is evaluated by checking whether the robot arm reaches the assigned target. Once the robot arm reaches the target, the decoder gets a positive reward +1, otherwise, it receives negative reward −1.

Table 2 shows the decoder performance over 4 days in terms of success rates. Each day corresponds to a separate experiment. In Day 1, the experiment has a total of 20 trials (10 A trials and 10 B trials). The overall success rate was 90%. Only the first trial for each target was incorrectly assigned.

**Figure 14:**
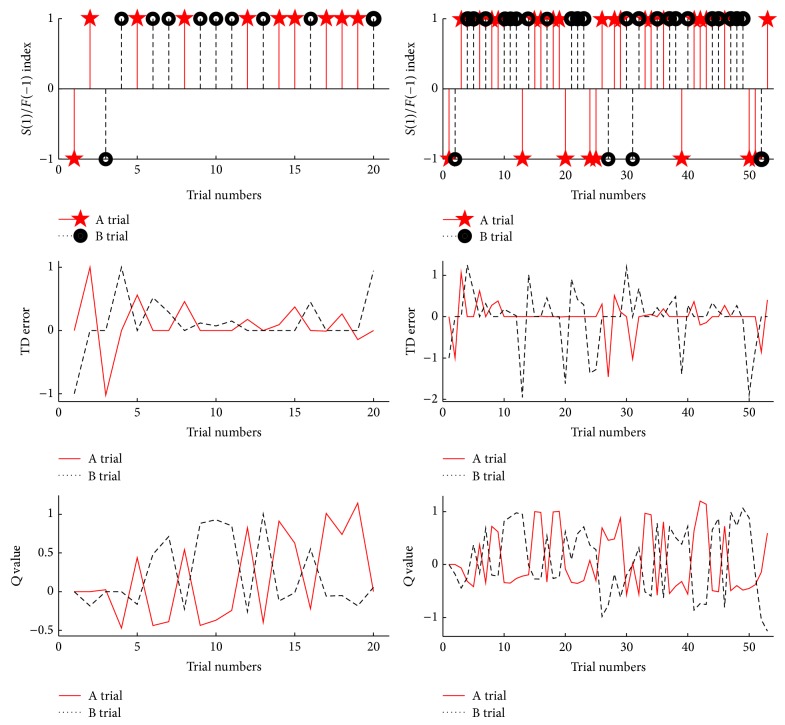
Performance of *Q*-learning via KTD in the closed loop RLBMI controlled by a monkey for Day 1 (left) and Day 3 (right); the success (+1) index and failure (−1) index of each trial (top), the change of TD error (middle), and the change of *Q*-values (down).

Note that at each day, the same experimental set up was utilized. The decoder was initialized in the same way at each day. We did not use pretrained parameters to initialize the system. To understand the variation of the success rates across days, we look at the performance of Day 1 and Day 3.  Figure 14 shows the decoder performance for the 2 experiments.

Although the success rate for Day 3 is not as high as Day 1, both experiments show that the algorithm learns an appropriate neural state to action map. Even though there is variation among the neural states within each day, the decoder adapts well to minimize the TD error, and the *Q*-values converge to the desired values for each action. Because this is a single step task and the reward +1 is assigned for a successful trial, it is desired for the estimated action value $\tilde{Q}$ to be close to +1.

It is observed that the TD error and *Q*-values oscillate. The drastic change of TD error or *Q*-value corresponds to the missed trials. The overall performance can be evaluated by checking whether the robot arm reaches the desired target (the top plots in Figure 14). However, this assessment does not show what causes the change in the system values. In addition, it is hard to know how the two separate intelligent systems interact during learning and how neural states affect the overall performance.

Under the coadaptation scenario in the RLBMI architecture, it is obvious that if one system does not perform properly, it will cause detrimental effects on the performance of the other system. If the BMI decoder does not give proper updates to the robotic device, it will confuse the user conducting the task, and if the user gives improper state information or the translation is wrong, the resulting update may fail even though the BMI decoder was able to find the optimal mapping function.

**Figure 15:**
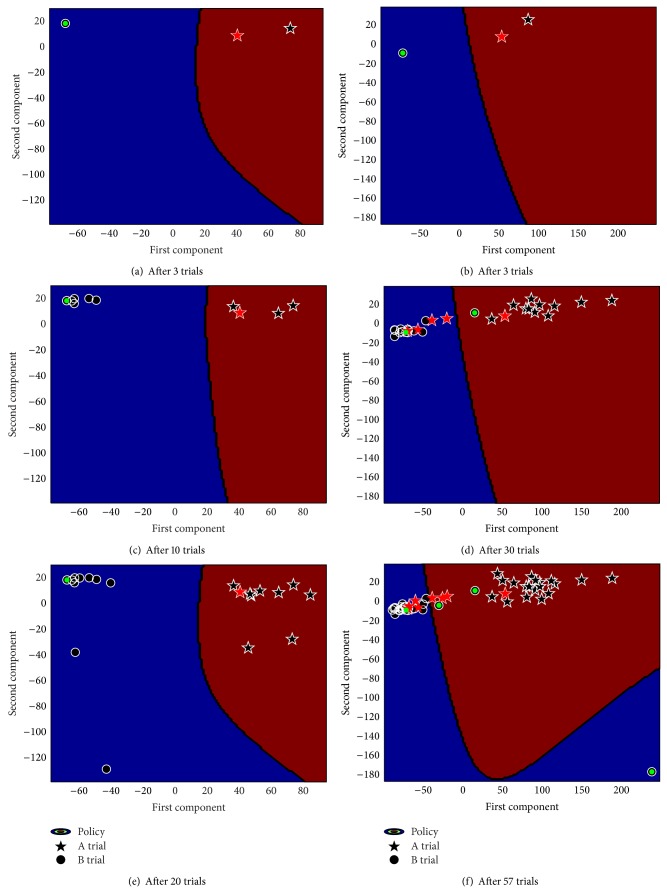
Estimated policy for the projected neural states from Day 1 (left) and Day 3 (right). The failed trials during the closed loop experiment are marked as red stars (missed A trials) and green dots (missed B trials).

Using the proposed methodology introduced in [36], we can observe how the decoder effectively learns a good state to action mapping, and how neural states affect the prediction performance.  Figure 15 shows how each participant (the agent and the user) influences the overall performance in both successful and missed trials, and how the agent adapts the environment. By applying principal component analysis (PCA), the high-dimensional neural states can be visualized in two dimensions using the first two largest principal components. In this two-dimensional space of projected neural states, we can visualize the estimated policy, as well.

We observe the behavior of two systems at the beginning, intermediate, and final stages of the experiment by using the neural states that have been observed as well as the learned decoder up to the given stage. It is evident that the decoder can predict nonlinear policies. Day 1 (left column in Figure 15) shows that the neural states from the two classes are well separable. It was noted during Day 3 that the monkey seemed less engaged in the task than in Day 1. This suggests the possibility that during some trials the monkey was distracted and may not have been producing a consistent set of neural outputs. We are also able to see this phenomenon from the plots (right column in Figure 15). We can see that most of the neural states that were misclassified appear to be closer to the states corresponding to the opposite target in the projected state space. However, the estimated policy shows that the system effectively learns. Note that the initially misclassified A trials (red stars in Figure 15(d) which are located near the estimated policy boundary) are assigned to the right direction when learning has been accomplished (Figure 15(f)). It is a remarkable fact that the system adapts to the environment online.

## 8. Conclusions

The advantages of KTD(*λ*) in neural decoding problems were observed. The key observations of this kernel-based learning algorithm are its capabilities for nonlinear function approximation and its convergence guarantees. We also examined the capability of the extended KTD algorithm (*Q*-KTD(*λ*)) in both open-loop and closed-loop reinforcement learning brain machine interface (RLBMI) experiments to perform reaching tasks.

In open-loop experiments, results showed that *Q*-KTD(*λ*) can effectively learn the brain state-action mapping and offer performance advantages over conventional nonlinear function approximation methods such as time-delay neural nets. We observed that *Q*-KTD(*λ*) overcomes main issues of conventional nonlinear function approximation methods such as local minima and proper initialization.

Results on closed-loop RLBMI experiments showed that the algorithm succeeds in finding a proper mapping between neural states and desired actions. Its advantages are that it does not depend on the initialization neither require any prior information about input states. Also, parameters can be chosen on the fly based on the observed input states. Moreover, we observed how the two intelligent systems coadaptively learn in an online reaching task. The results showed that KTD is powerful for practical applications due to its nonlinear approximation capabilities in online learning.

The observation and analysis of KTD(*λ*) give us a basic idea of how this algorithm behaves. However, in the case of *Q*-KTD(*λ*), the convergence analysis remains challenging since *Q*-learning contains both a learning policy and a greedy policy. For *Q*-KTD(*λ*), the convergence proof for *Q*-learning using temporal difference (TD)(*λ*) with linear function approximation in [37] can provide a basic intuition for the role of function approximation on the convergence of *Q*-learning.

## Figures and Tables

**Table 1:** Average success rates of *Q*-KTD in open-loop RLBMI.

Epochs	1	2	3	4	5	6	7
2 target	0.44	0.96	0.99	0.99	0.97	0.99	0.99
4 target	0.41	0.73	0.76	0.95	0.99	0.99	0.99
8 target	0.32	0.65	0.79	0.89	0.96	0.98	0.98

**Table 2:** Success rates of *Q*-KTD in closed-loop RLBMI.

	Total trial numbers (total A, B trial)	Success rates (%)
Day 1	20 (10, 10)	90.00
Day 2	32 (26, 26)	84.38
Day 3	53 (37, 36)	77.36
Day 4	52 (37, 35)	78.85
